# Structure-based insights into fluorogenic RNA aptamers

**DOI:** 10.3724/abbs.2024142

**Published:** 2024-08-16

**Authors:** Qianqian Song, Xiaoqing Tai, Qianyu Ren, Aiming Ren

**Affiliations:** 1 Life Sciences Institute Second Affiliated Hospital of Zhejiang University School of Medicine Zhejiang University Hangzhou 310058 China; 2 Agricultural College Yangzhou University Yangzhou 225009 China

**Keywords:** fluorogenic RNA aptamers, tertiary structures, base quadruples, base triples, fluorescence activation mechanisms, G-quadruplex

## Abstract

Fluorogenic RNA aptamers are *in vitro-*selected RNA molecules capable of binding to specific fluorophores, significantly increasing their intrinsic fluorescence. Over the past decade, the color palette of fluorescent RNA aptamers has greatly expanded. The emergence and development of these fluorogenic RNA aptamers has introduced a powerful approach for visualizing RNA localization and transport with high spatiotemporal resolution in live cells. To date, a variety of tertiary structures of fluorogenic RNA aptamers have been determined using X-ray crystallography or NMR spectroscopy. Many of these fluorogenic RNA aptamers feature base quadruples or base triples in their fluorophore-binding sites. This review summarizes the structure-based investigations of fluorogenic RNA aptamers, with a focus on their overall folds, ligand-binding pockets and fluorescence activation mechanisms. Additionally, the exploration of how structures guide rational optimization to enhance RNA visualization techniques is discussed.

## Introduction

RNA is one of the most fundamental biomacromolecules in life and plays a crucial role in diverse biological processes, such as genetic information translation, gene expression regulation, and maintenance of cell functionality [ 1‒ 4]. The visualization of RNA localization and dynamics with high spatial and temporal resolution is essential for investigating their functions, mechanisms and interactions in biology. Fluorescent proteins, especially green fluorescent protein (GFP), have revolutionized the spatiotemporal localization of proteins and the investigation of protein interactions both *in vivo* and *in vitro* [ 5‒ 8]. However, intrinsic fluorescent RNAs comparable to GFP have not been identified until now. Current techniques used for the dynamic detection of RNA molecules include fluorescence *in situ* hybridization (FISH) [ 9, 10], molecular behavior technology [11], and RNA hairpin methods [ 12‒ 18]. While effective, FISH requires cell fixation and cannot be used for live-cell imaging [ 9, 10]. Molecular behavior technology allows spatiotemporal imaging of RNA molecules in living cells but is limited by false-positive signals [11]. Alternatively, RNAs of interest can be labelled with naturally occurring RNA hairpins along with their specific binding proteins ( *e*. *g*., MS2-MCP [12], PP7-PCP [14], λN _22_-boxB [15] and gRNA-dCas [ 16‒ 18]) fused to fluorescent proteins. Unfortunately, this technique suffers from the high background fluorescence of unbound fluorescent proteins. Recently, RNA-based fluorogenic aptamers have been used for advanced live-cell RNA imaging [ 19‒ 28]. These fluorogenic RNA aptamers, which evolved *in vitro* through Systematic Evolution of Ligands by Exponential Enrichment (SELEX) technology, can specifically bind to their cognate fluorogenic dyes and significantly activate their fluorescence [ 29‒ 32].

The Malachite Green aptamer was the first fluorescent RNA aptamer, originally developed in 2003 [ 33, 34]. In 2011, the Jaffrey group synthesized a series of fluorogenic HBI analogues and selected an RNA mimic of GFP called Spinach, marking a critical breakthrough in the development of fluorogenic RNA aptamers [35]. Since then, various approaches have been employed to fine-tune and improve the properties of Spinach and its cognate fluorophore molecules, leading to the identification of related aptamers such as Broccoli [36], Corn [37], Beetroot [38], Chili [39] and Squash [40] aptamers. In addition, multiple other fluorescent RNA aptamer systems have been isolated and characterized, including cyanine dye-based aptamers [ 41‒ 44], contact quenching-based aptamers [ 45‒ 52], and spirolactonization-based aptamers [ 53, 54]. Recently, two novel fluorogenic aptamers, Pepper and Clivia, which feature high cellular brightness, good photostability and multiple spectral properties, have been developed, greatly enriching the available toolbox for RNA imaging [ 55‒ 57] .

A structure-based investigation of these fluorescent RNA aptamers is essential for understanding the mechanisms of ligand recognition and fluorescence activation, providing robust structural guidance for their effective application both *in vivo* and *in vitro*. In this review, we focus on the structural research progress of these fluorescent RNA aptamers. By comparing the overall fold and composition of the fluorophore binding sites among these fluorogenic RNA aptamers, we aim to elucidate the mechanisms underlying ligand recognition and fluorescence activation. Additionally, we discuss how structural studies advance the rational optimization of RNA aptamers and the modification of fluorophores with improved photophysical properties, facilitating their application in biosensing and bioimaging.

## The Malachite Green aptamer

**Figure 1:**
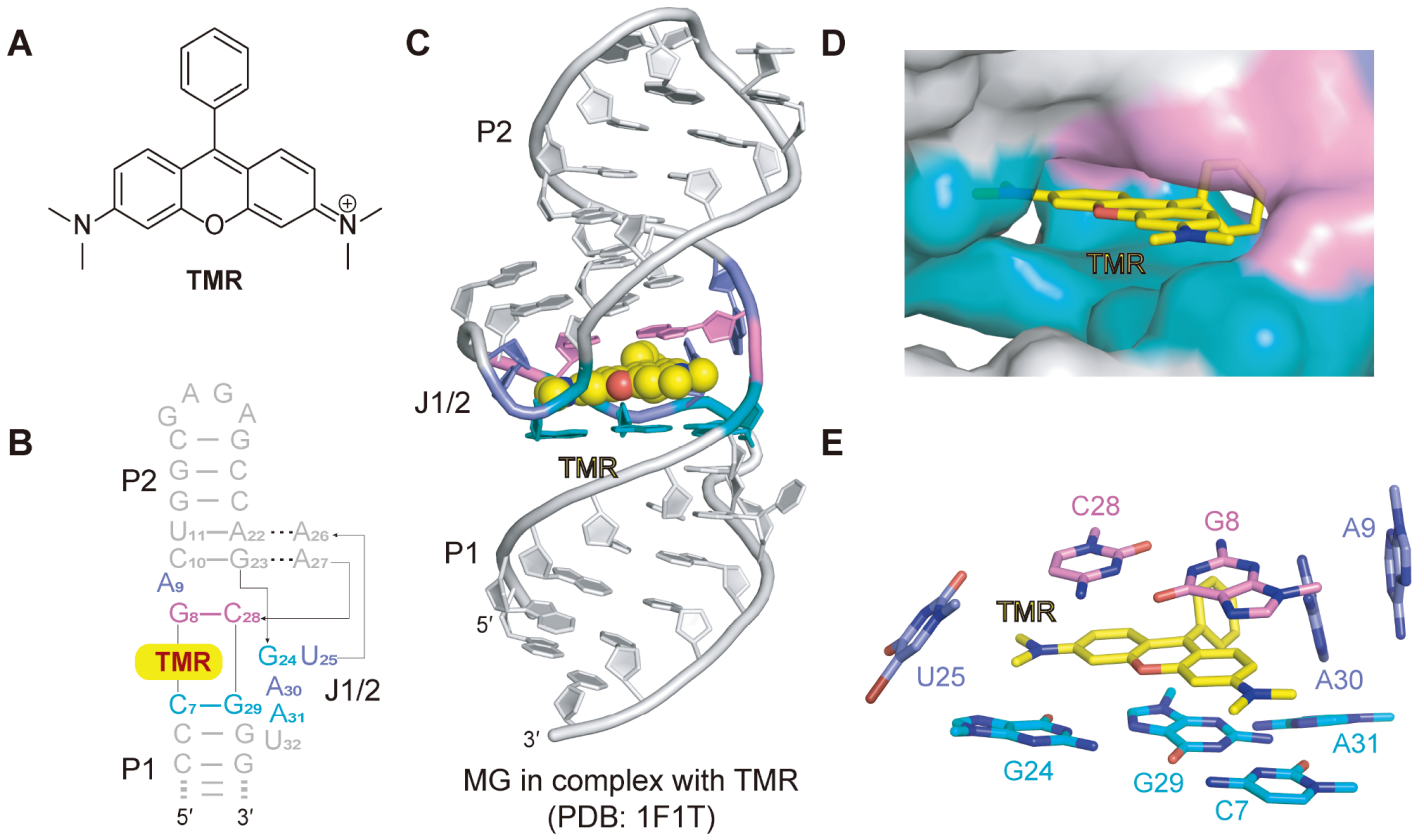
Figure 1 Structural topology and TMR-binding pocket of the malachite green (MG) aptamer (A) Chemical structure of TMR. (B,C) Schematic and cartoon representation of the MG aptamer based on the tertiary structure of the MG aptamer in complex with the ligand TMR. The TMR is shown in balls in panel (C). (D) Surface representation of the binding pocket of the MG aptamer with the ligand TMR shown as sticks. (E) Stack representation of the TMR binding pocket of the MG aptamer. The TMR is sandwiched between two bases above (C28-G8) and four bases below (G24-G29-A31-C7) while being surrounded by A9, A30, and U25 on both sides. Panels (B‒E) are depicted on the basis of PDB 1F1T.

The malachite green (MG) aptamer was the first fluorescent RNA aptamer, which was originally isolated by Wilson *et al*. [33] in 1999 through *in vitro* selection for its use in laser-assisted cleavage and inactivation of RNA transcripts. In 2003, Tsien *et al*. [34] reported that the MG aptamer not only selectively binds to the triphenylmethane dye MG but also switches on its fluorescence approximately 2400-fold. Subsequent characterization of the MG aptamer demonstrated that the aptamer has a greater affinity for the rigid planar analogue tetramethylrosamine (TMR; Figure 1A), with a dissociation constant *K*_d_ of 40 nM, than for the nonplanar and rotationally mobile MG (800 nM) [58].

The crystal structure of the MG aptamer in complex with TMR was first determined by Wilson’s group in 2000, with a resolution of 2.8 Å [58]. Dieckmann’s group subsequently utilized NMR spectroscopy to solve the tertiary structure of the MG aptamer bound to MG [ 59, 60]. In the following description, we discuss the crystal structure of the MG aptamer in complex with TMR (PDB: 1F1T), which adopts a rod-like compact helical scaffold (‘I-shape’) [ 58‒ 60]. Stems P1 and P2 exhibit coaxial stacking mediated by junctional segments, and the ligand-binding pocket is positioned at the center of the whole structure ( Figure 1B,C). A base quadruple (G24-G29-A31-C7) and a Watson-Crick base pair (C28-G8) constitute the floor and ceiling of the binding pocket and sandwich the ligand TMR on both sides ( Figure 1D,E). Additionally, two base triples, C10-G23-A27 and U11-A22-A26, form the top of the binding pocket and do not interact directly with the bound ligand ( Figure 1B,C). On the right side of the ligand-binding pocket, the base A30 stacks directly against the phenyl ring of TMR and is further stabilized by its stacking with the base A9 ( Figure 1E).

Notably, the solution structures of the aptamer in complex with TMR and MG revealed minor differences in the stacking arrangement within the ligand-binding site [ 58, 59]. Compared with TMR, which adopts a planar orientation, MG is bound to the aptamer in a slightly twisted conformation, resulting in the loss of stacking interactions between the dimethylaniline ring of MG and RNA [ 58, 59]. These structural differences are consistent with the reduced stability and affinity of the MG-RNA complex.

To date, the MG aptamer has been developed as a fluorescent biosensor for the detection of small molecules and engineered as a split fluorescent probe for nucleic acid detection [ 61, 62]. However, the widespread application of MG in cellular imaging is limited by its cell toxicity, which is induced by the generation of free radicals upon excitation [63]. Nevertheless, the MG aptamer has introduced potential for the development of genetically encoded fluorescent RNA aptamers with high affinity and fluorescence brightness, offering promising applications for various studies.

## HBI Analogues with Spinach and Related Aptamers

**Figure 2:**
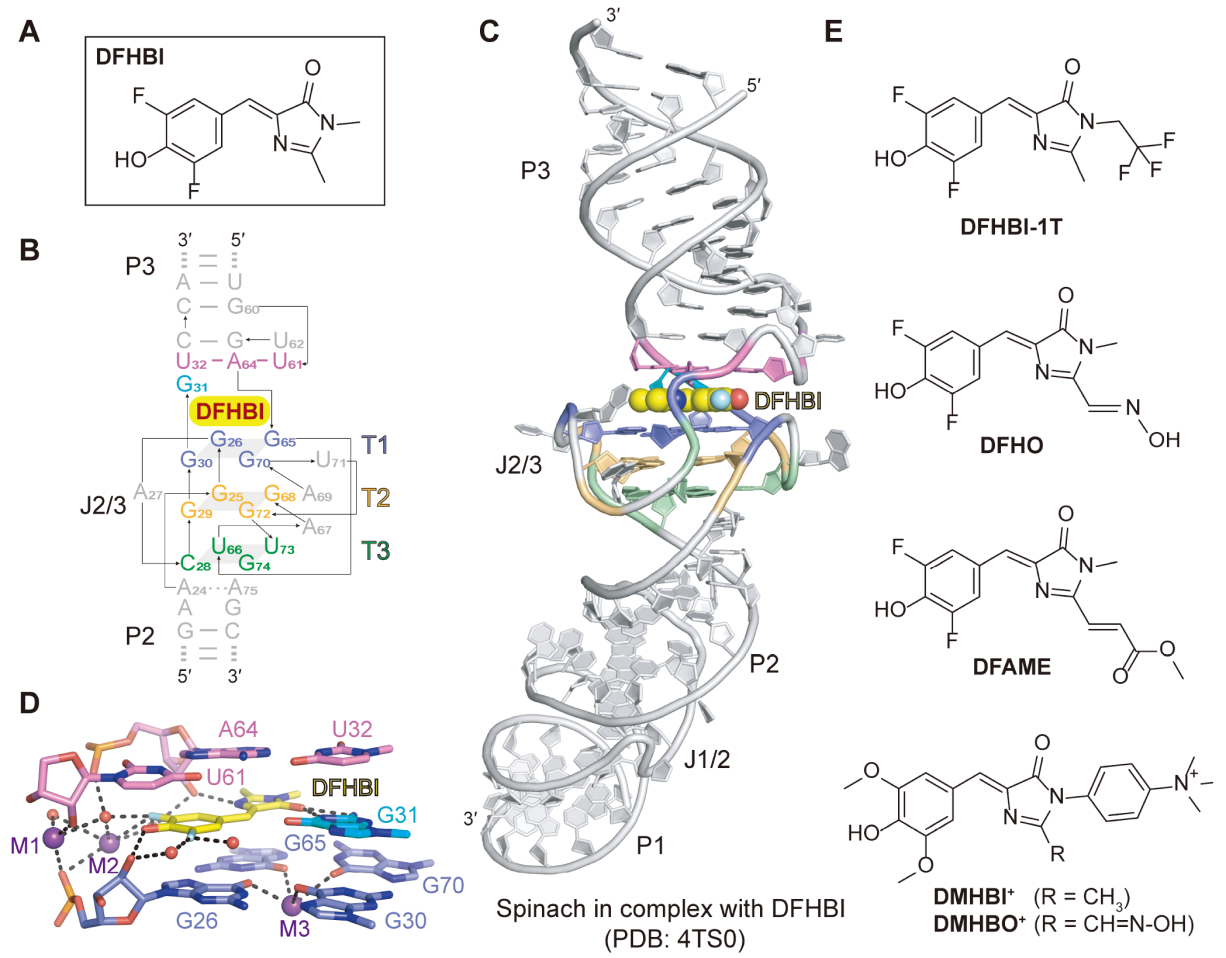
Figure 2 Structural topology and DFHBI-binding pocket of the Spinach aptamer (A) Chemical structure of DFHBI. (B) Close-up view of the schematic secondary structure of the Spinach aptamer with emphasis on the junction region J2/3, stem P2 and stem P3. The color coding of the sequence is the same as that of the tertiary structure in panel (C). (C) Cartoon representation of the tertiary structure of Spinach aptamer with the bound ligand DFHBI shown in balls. (D) Stack representation of the binding pocket of Spinach aptamer with the bound ligand DFHBI. The ligand DFHBI intercalates into the ligand-binding pocket, with the bottom layer formed by G26 and G65 and the top layer comprising the U32-A64-U61 base triple. Several K+ ions (M1, M2 and M3) and water molecules have been identified to participate in the DFHBI-binding pocket of the Spinach aptamer. (E) Chemical structures of DFHBI-1T, DFHO, DFAME, DMHBI + and DMHBO+. Panels (B‒E) are depicted on the basis of PDB 4TS0.

In 2011, the Jaffrey group developed RNA mimics of GFP, called Spinach, that bind to and activate the fluorescence of [(Z)-4-(3,5-difluoro-4-hydroxybenzylidene)-1,2-dimethyl-1H-imidazol-5(4H)-one] (DFHBI; Figure 2A) [35]. DFHBI is a small-molecule mimic of the intrinsic chromophore of GFP, known as 4-hydroxybenzylidene imidazolinone (HBI). Like HBI, DFHBI has low or no fluorescence but exhibits strong green fluorescence upon binding to Spinach both *in vitro* and in living cells. Importantly, DFHBI has no cytotoxicity or phototoxicity. Furthermore, the phenolate form of the fluorophore has a high extinction coefficient and quantum yield, making it an excellent choice for identifying fluorogenic RNA aptamers that bind to and switch on its fluorescence [ 8, 35].

The crystal structure of the Spinach in complex with DFHBI (PDB: 4TS0) reveals a long coaxial helical stack composed of three stems (P1, P2 and P3) connected by two irregular junctions (J1/2 and J2/3) ( Figure 2 B,C) [ 64, 65]. The J2/3 junction organizes a three-tetrad quadruplex consisting of two G-quartets (T1 and T2) stacked above a mixed-base tetrad (T3), which is stabilized by two potassium ions ( Figure 2B,C). In the DFHBI-binding pocket, the bottom layer is formed by G26 and G65 of the top G-quartet (T1), whereas the top layer comprises the U32-A64-U61 base triple ( Figure 2D). This precise arrangement effectively constrains the imidazolone group and phenyl rings of the fluorophore, promoting a coplanar conformation. The N3 atom and phenolic oxygen of DFHBI form hydrogen bonds with the ribose 2′-OH of G26 and A64, respectively ( Figure 2D). Additionally, the unpaired G31 residue on the side of the DFHBI-binding pocket forms hydrogen bonding interactions with the carbonyl oxygen of the ligand ( Figure 2D). Notably, several K ^+^ ions (M1 and M2) and water molecules also participate in the DFHBI-binding pocket of the Spinach aptamer. M2 is located on the side of the ligand and forms one hydrogen bond with the phenolic oxygen of DFHBI ( Figure 2D). In addition, coordination interactions are established between the fluorine atoms of DFHBI and water.

Owing to the cell permeability, noncytotoxicity and nonphototoxicity of DFHBI, Spinach has been successfully utilized for tagging and imaging RNA expression, localization, and transport, as well as for the design of genetically encoded sensors to detect and monitor various essential metabolites in living systems [ 35, 66‒ 72]. However, challenges have limited its broad application, such as the misfolding tendency of Spinach aptamer and poor photostability [ 66, 73‒ 75]. On the basis of the crystal structure of the Spinach-DFHBI complex, a miniaturized version called “Baby Spinach” (50 nucleotides) was generated with improved folding properties [73]. To further improve the thermal stability and folding of Spinach in living cells, Spinach2 was developed through systematic mutagenesis [66]. Another enhanced version of Spinach, designated iSpinach, was selected using random mutagenesis and high-throughput screening by microfluidic-assisted *in vitro* compartmentalization, as reported by the Ryckelynck’s group [76]. The overall structure and DFHBI-binding pocket of iSpinach are very similar to those of the original Spinach aptamer [77]. A combination of the standard SELEX procedure and directed evolution by fluorescence-activated cell sorting (FACS) produces the broccoli aptamer, which exhibits improved brightness and binds to [(Z)-4-(3,5-difluoro-4-hydroxybenzylidene)-2methyl-1-(2,2,2-trifluoroethyl)-1H-imidazol-5(4H)-one] (DFHBI-1T; Figure 2E), a modified version of DFHBI [ 36, 78]. However, the Spinach-DFHBI complex easily suffers photobleaching under continuous large-dose light irradiation due to light-induced isomerization of DFHBI from the *cis* to the *trans* form, which limits its wide use [ 74, 75].

To increase the photostability and folding of fluorescent Spinach and expand the spectral properties of related aptamers, the Jaffery group designed a series of DFHBI analogue fluorophores. One of these, 3,5-difluoro-4-hydroxybenzylidene imidazolinone-2-oxime (DFHO), is based on the chromophore found in DsRed and other red fluorescent proteins ( Figure 2E). Compared with DFHBI, DFHO contains an additional N-hydroxy imine substituent at the C2 position, which extends the π-conjugation of the fluorophore and potentially improves RNA-mediated photostability. Through SELEX technology, a novel RNA aptamer named Corn, which specifically binds to and activates the yellow fluorescence of DFHO, was developed [37]. Another fluorophore, 3,5-difluoro-4-hydroxybenzylidene imidazolinone-2-acrylate methyl (DFAME), was generated by substituting the hydroxamic acid in DFHO with methyl acrylate ( Figure 2E) [38]. Compared with DFHO, DFAME contains a more extended π-electron conjugation system, resulting in redshifted fluorescence emission and excitation. Beetroot was developed to bind to and induce the fluorescence of DFAME via SELEX [38]. On the basis of the chemical structure of the HBI chromophore and Spinach system scaffold, the Höbartner group performed structural-guided truncation and sequence optimization of the 13-2 RNA aptamer, a variant of Spinach that binds to dimethoxy-HBI (DMHBI; Figure 2E), and identified a large Stokes shift fluorescent aptamer named Chili [39]. Notably, the tertiary structures of Corn, Beetroot and Chili RNA aptamers all contain G-quadruplex structural elements in their core domains [ 79‒ 81]. Fine-tuning chromophores through chemical modifications provides new insights into the optimization of fluorescent RNA aptamers with improved photophysical properties.

In 2021, the Jaffrey’s laboratory successfully engineered a naturally occurring well-folded adenine riboswitch into a fluorescent RNA aptamer named Squash. Squash binds to and strongly activates the fluorescence of DFHBI-1T and DFHO [40]. The stable overall scaffold and expanded ligand-binding pocket endow Squash with improved intracellular folding and enhanced photostability, offering potential in designing ratiometric biosensors for imaging metabolite levels [ 40, 82] .

## Cyanine Dye-based Fluorogenic RNA Aptamers

**Figure 3:**
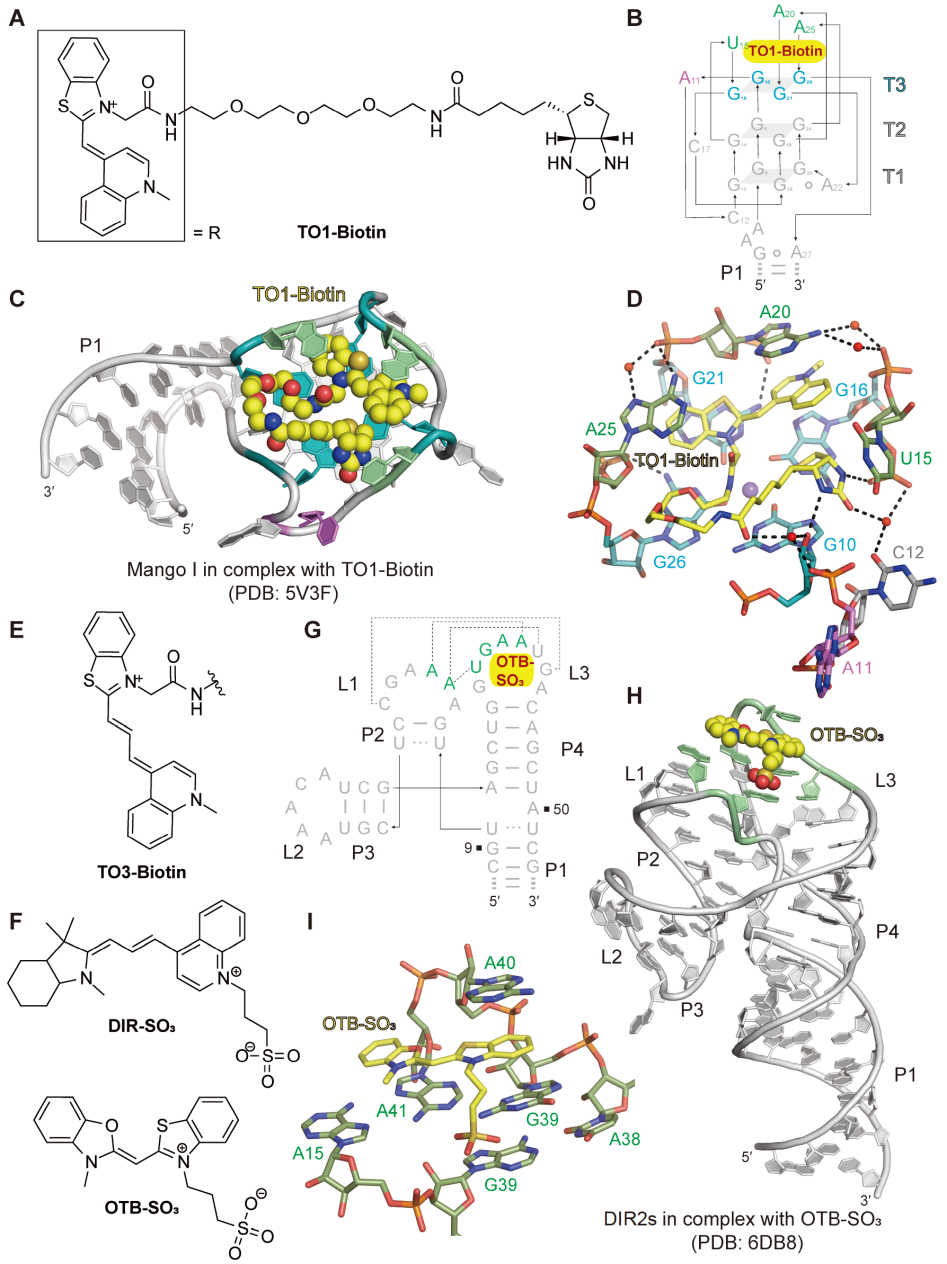
Figure 3 Structural topology and cyanine-based fluorophore-binding pocket of the Mango I aptamer and DIR2s aptamer (A) Chemical structure of TO1-Biotin. (B) Fose-up view of the schematic secondary structure of the Mango I aptamer with emphasis on the three-tiered G-quadruplex (T1, T2, T3) and several loops containing key bases involved in forming the binding pocket. The color is coded in panel (C). (C) Cartoon representation of the tertiary structure of the Mango I aptamer with the ligand TO1-Biotin shown in balls. (D) Stack representation of the binding pocket of the Mango I aptamer with the ligand TO1-Biotin. The loops (A11, C12, U15, A20, A25) stabilize each other through direct or water-mediated hydrogen bonds to form a binding pocket, whereas the hydrogen bonds between G10 and U15 with the biotin portion of the ligand further stabilize the binding of TO1-Biotin. (E) Chemical structure of TO3-Biotin. The omitted PEG linker and biotin moieties are the same as those of TO1-Biotin in panel A. (F) Chemical structures of DIR-SO3 and OTB-SO3. (G,H) Tertiary structure and schematic secondary structure of the DIR2s aptamer with an emphasis on the three stem loops. (I) Stack representation of the binding pocket of the DIR2s aptamer with the ligand OTB-SO3. Panels (B‒D) are depicted on the basis of PDB 5V3F, and panels (G‒I) are depicted on the basis of PDB 6DB8.

Thiazole orange TO is a canonical fluorophore with an asymmetric cyanine skeletal structure that exhibits very low fluorescence in aqueous solution. Typically, the fluorescence of TO is significantly enhanced when the monomethine bridge connecting the two heterocycles is rigid through nonspecific insertion into double-stranded helical nucleic acids [83]. Adding substituents to the benzothiazole heterocycle in TO significantly reduces its nonspecific insertion. The derivative TO1-Biotin ( Figure 3A) was synthesized for screening high-affinity fluorescence turn-on aptamers, leading to the successful selection of the Mango I RNA aptamer, which has a 23 nt core sequence ( Figure 3B,C). Mango I has a high binding affinity for TO1-Biotin, with a *K*_d_ of 3.6 nM and an approximately 1100-fold increase in fluorescence. This enables imaging of Mango I-TO1-Biotin in microinjected live *C*. *elegans*, highlighting its potential applications in the visualization of cellular RNAs [42].

The crystal structure of Mango I in complex with TO1-Biotin (PDB: 5V3F) reveals a relatively simple RNA structure ( Figure 3B,C). A three-tiered G-quadruplex (T1, T2, and T3) containing three antiparallel guanine residues (G16, G21, and G26 in the T3 plane) is connected by a GAAA tetraloop to an A-form duplex [84]. The three G-quadruplexes of Mango I are connected by six loops (A11, C12, U15, A20, and A25), with A22 and T1 lying in the same plane, resulting in T1 being extended into a pentagon. The interaction between U15, A20, and A25 causes the RNA chain to flip at the top of T3 and form the binding pocket for TO1-Biotin ( Figure 3B‒D). The stabilization of three consecutive stacked G-quadruplexes is also facilitated by coordination with K ^+^, which is a highly characteristic feature of the G-quadruplex structural motif ( Figure 3D). Additionally, the complete fluorescent group, comprising TO1, biotin, and the PEG connectors that bind them together, extensively interacts with RNA. In the complex structure of mango I/TO1-biotin, TO1-Biotin adopts a cyclic conformation, with biotin and methylquinoline heterocycles closely aligned ( Figure 3D). The three heterocycles of TO1-Biotin stack with three nucleotides in the RNA loop: methylquinoline with A20, benzothiazole with A25, and biotin with U15 ( Figure 3D). Multiple hydrogen bonds stabilize the binding of TO1-Biotin with RNA, including direct hydrogen bonds (the ureido nitrogens of biotin with the 2′-OH of G10 and a phosphate oxygen of U15) and water-mediated hydrogen bonds (the carboxyl group of biotin and the phosphates of G10 and A11, the carbonyl group of the head group and the phosphate of U15, and the O2 carbonyl group of C12) [84].

After the emergence of the Mango I aptamer, Autour *et al*. [44] employed a competitive ligand binding method combined with microfluidic selection to rescreen the original round 12 Mango I library (R12). As a result, they successfully obtained three new aptamers, namely, Mango II, III, and IV. Compared with the original Mango I aptamer, these new variants exhibit significant improvements in their fluorescence properties and reduced salt dependency. Compared with Mango I, Mango II has a greater binding affinity for TO1-Biotin, whereas Mango III and IV have slightly lower affinities. Among these three novel aptamers, Mango II and IV have a relatively high degree of sequence similarity with Mango I. Structure-based investigations revealed that Mango II, III, and IV also possess a G-quadruplex-tiered structure [ 85‒ 87]. TO3-Biotin, a derivative of TO1-Biotin, has a similar affinity with Mango II, with a *K*_d_ value of 3.1 nM, compared with the 8 nM *K*_d_ value for TO1-Biotin [85] ( Figure 3E).

Dimethylindole red (DIR) is another type of cyanine dye ( Figure 3F) designed with a dimethylindole heterocycle and anionic propylsulfonic acid substituents to reduce nonspecific binding with nucleic acids. After 15 rounds of SELEX targeting DIR, the DIR-Apt1 aptamer was yielded [41]. The DIR2 aptamer, 57 nt in length, enhances the fluorescence of the DIR dye and oxazole thiazole blue (OTB) by 50-fold and 53-fold, respectively ( Figure 3F–G). Owing to their distinct spectral characteristics, DIR2s aptamer enable dual-color excitation in the red (DIR) and blue (OTB) regions, facilitating effective imaging of exogenous RNA both intra- and extracellularly [43]. Sandip A. Shelke and colleagues utilized the Fab BL3-6 antibody as an RNA crystallization chaperone [88] to solve the structure of the DIR2s aptamer in complex with OTB-SO _3_ (PDB: 6DB8) [89]. The crystal structure revealed a tuning fork-like fold composed of two short stem loops and one long helix, with OTB-SO _3_ binding at the top terminal ( Figure 3G,H). OTB-SO _3_ is encapsulated in a stacking sandwich with the lower three purine bases (G39, A41 and A15) and the upper single-adenine nucleobase (A40), in which the benzothiazolium and benzoxazole rings adopt a coplanar conformation ( Figure 3I). The propylsulfonate side chain forms hydrogen bonds with G39 within the base triple ( Figure 3I). Notably, the DIR2 aptamer tends to dimerize, which affects its broader application.

## The Bright Green Pepper Aptamer

**Figure 4:**
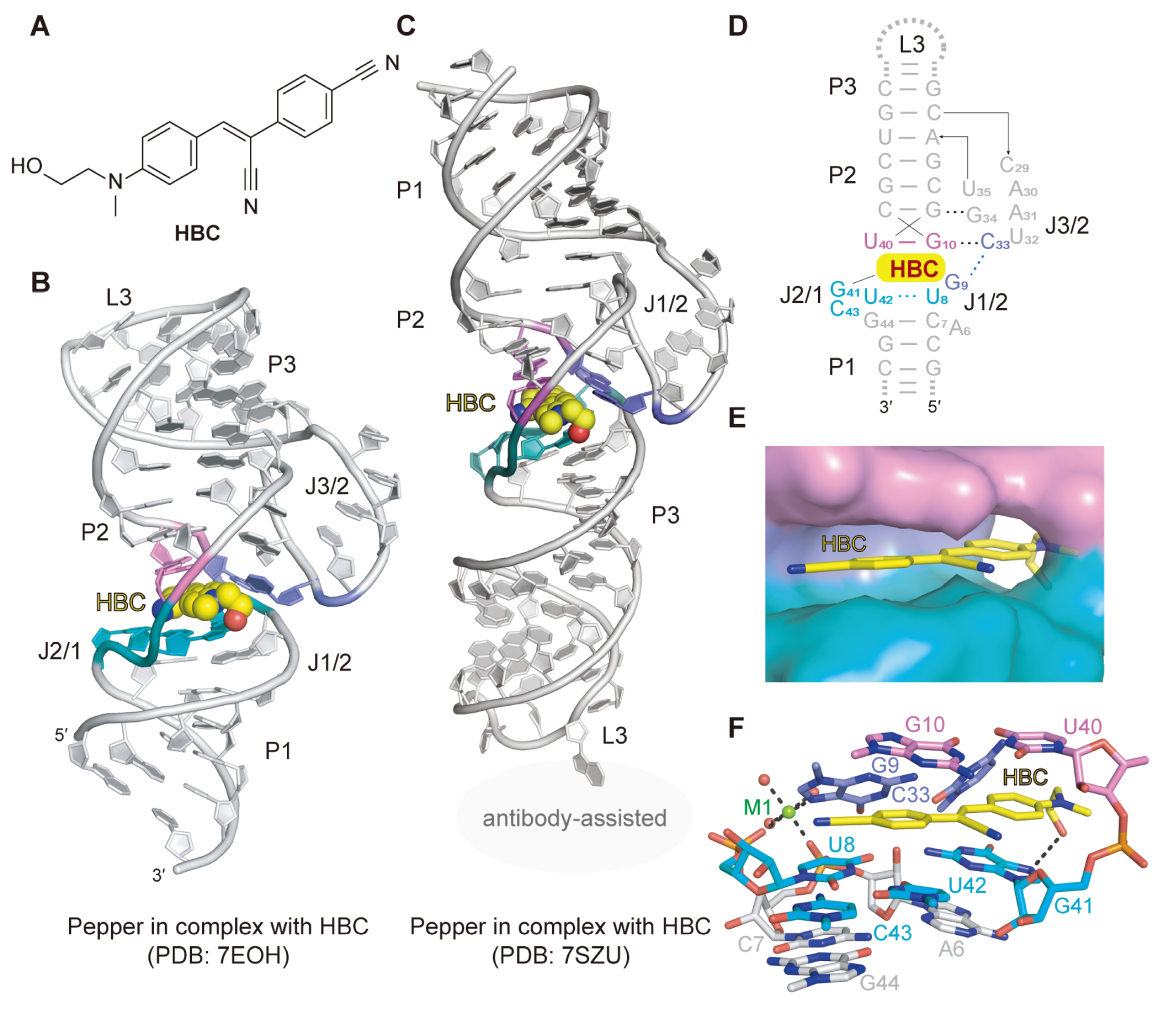
Figure 4 Structural topology and HBC-binding pocket of the Pepper aptamer (A) Chemical structure of HBC. (B) Cartoon representation of the tertiary structure of the Pepper aptamer with the bound ligand HBC shown in balls (PDB: 7EOH). (C) Tertiary structure of the Pepper aptamer in complex HBC solved with the assistance of an antibody (PDB:7SZU). (D) Schematic secondary structure of the Pepper aptamer on the basis of the tertiary structure. The color coding of the sequence is the same as that in panel (B). (E) Surface representation of the binding pocket of the Pepper aptamer with the ligand HBC shown as sticks. HBC intercalates into the ligand-binding pocket, resulting in stacking and burial of the chromophore. (F) Sticks representation of the binding pocket of the Pepper aptamer with the ligand HBC. HBCs are encapsulated in a four-sided box arrangement. The top and bottom faces are constituted by one noncanonical base pair, G10-U40, and the non-G-quadruplex base quadruple, G41-U42-C43-U8, respectively. G9 and C33 and the linked phosphate between U40 and G41 comprise the faces on the left-hand and right-hand sides. Panels (D‒E) are depicted on the basis of PDB 7EOH.

Recently, Yang and coworkers selected and characterized a novel fluorogenic RNA aptamer termed Pepper, which can selectively bind to and activate a new synthetic dye, [4-((2-hydroxyethyl)(methyl)amino)-benzylidene]-cyanophenylacetonitrile (HBC) ( Figure 4A), to emit strong green fluorescence [55]. Compared with previously existing green fluorescent RNA aptamers, the Pepper-HBC complex displays one order of magnitude greater cellular brightness and one or two orders of magnitude greater fluorophore affinity. These excellent photophysical properties make Pepper an ideal tool for labelling and imaging diverse RNAs, enabling investigations into their complex spatiotemporal dynamics and biological functions within living cells [55].

The crystal structure of Pepper bound to HBC (PDB: 7EOH), solved in 2021 [56], and the antibody-assisted cocrystallization structure of Pepper in complex with HBC (PDB: 7SZU), solved in 2022 [90], both reveal that Pepper folds in a monomeric, non-G-quadruplex tuning-fork-like helical scaffold, with three helices (stem P1, P2 and P3) coaxially stacked, mediated by junctional segments (J1/2, J2/1 and J3/2) ( Figure 4B,C). In both structures, the ligand-binding pocket is located in the middle of the structure with the same nucleotide alignment ( Figure 4B,C). For further discussion, we will focus on one structure (PDB: 7EOH) to present the structural details. As shown in Figure 4D, HBC is bound at the intersection of stem P2 and the junctional regions J1/2 and J2/1, capped by the bulge region J3/2. The surface representation of the ligand-binding pocket shows that the two phenyl ring moieties of HBC are positioned in a near-planar conformation, with the hydroxyethyl moiety of HBC inserting inwardly into the binding pocket ( Figure 4E). Within the binding pocket, the top is formed by the wobble base pair G10-U40 at the terminus of stem P2, whereas the bottom is constituted by the non-G-quadruplex base quadruple G41-U42-C43-U8 ( Figure 4F). This arrangement provides a platform to accommodate the bound ligand HBC. The terminal residues C33 in J3/2 and G9 in J1/2 tightly bracket the bound HBC molecule from the side, further anchoring it within the binding pocket ( Figure 4E). Notably, the terminal hydroxyl group of HBC forms a hydrogen bond with N7 of G41 at J2/1, which is important for maintaining the high ligand specificity of the Pepper aptamer. One magnesium ion is observed in the vicinity of the HBC-binding pocket, which forms direct coordination bonds with N7 of G9, the phosphate between A6 and C7 and the phosphate between C7 and U8 ( Figure 4E). The structures of the Pepper aptamer bound to HBC analogues indicate that modifications of HBC are feasible in the binding pocket, opening possibilities for rational design of fluorophore molecules to generate a broad spectral range and high quantum yield. In summary, the comprehensive analysis of the overall structure of the Pepper-HBC complex and binding pocket provides a robust structural foundation for the enhanced and efficient utilization of the Pepper aptamer both *in vivo* and *in vitro*.

## Clivia Aptamer with Large Stokes Shift

**Figure 5:**
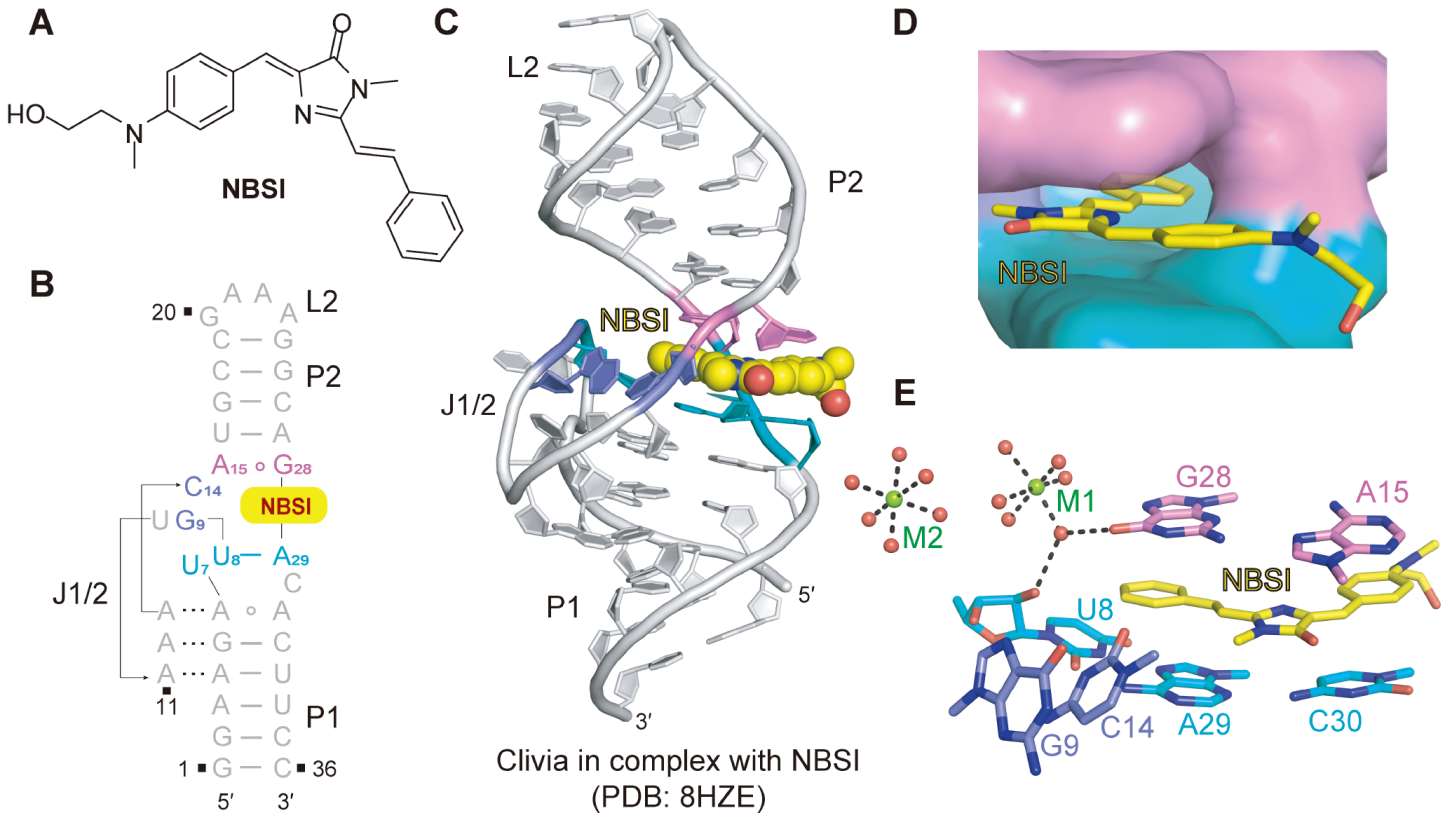
Figure 5 Structural topology and NBSI-binding pocket of the Clivia aptamer (A) Chemical structure of NBSI. (B) The schematic secondary structure of the Clivia aptamer is depicted on the basis of the tertiary structure. The color coding of the sequence is the same as that in panel (C). (C) Cartoon representation of the tertiary structure of the Clivia aptamer with the ligand NBSI shown in balls. NBSI is located at the center of the overall structure and intercalates between two helical segments of the Clivia aptamer. (D) Surface representation of the binding pocket of the Clivia aptamer with the ligand NBSI shown as sticks. (E) Stack representation of the binding pocket of the Clivia aptamer with the ligand NBSI. The planar moiety of the ligand NBSI is surrounded by three groups of consecutive junction residues: U8-G9, C14-A15 and G28-A29-C30. A15 and G28 form one base pair and stack above NBSI. U8, A29 and C30 form one base triple and stack below NBSI. G9 and C14 are stacked on each other and bracket the side of the NBSI. Two fully hydrated Mg2+ ions (shown in balls) were identified around the NBSI-binding pocket. Panels (B‒E) are depicted in PDB 8HZE.

Inspired by the work of RNA mimics of fluorescent proteins, a new fluorophore termed 4-(N-(2-hydroxyethyl)(methyl))benzylidene-3-methyl-2-styryl-3,5-dihydro-4H-imidazol-4-one (NBSI; Figure 5A) was synthesized. The design of fluorophores is based on the naturally occurring fluorophore in red fluorescent proteins, which incorporates a dialkylamino group as the electron donor and a styryl group to increase molecular flexibility and intramolecular charge transfer [57]. NBSI exhibits negligible fluorescence in solution but shows strong fluorescence upon binding to its cognate aptamer, named Clivia. Compared with other fluorogenic RNA aptamers, Clivia is a compact monomer consisting of only 36 nucleotides ( Figure 5B). The excellent photophysical characteristics of Clivia, such as low background fluorescence, enhanced cellular brightness, and large Stokes shifts (large spectral shifts between excitation and emission peaks) up to 108 nm, provide advantages for the simultaneous visualization and tracking of various RNA molecules in live cells [57].

The complex structure of Clivia/NBSI (PDB: 8HZE) folds into a single coaxial helix containing two duplexes, P1 and P2, which are connected by the zipped junction region J1/2 ( Figure 5B,C) [91]. Consecutive nucleotides from G9 to C14 within the internal loop J1/2 extrude from the helix and interact with the minor groove of stem P1 ( Figure 5B,C). The surface representation shows that NBSI adopts a near-planar conformation stabilized by the planar and compact ligand-binding pocket at the central junction of the Clivia aptamer structure ( Figure 5D). The major aromatic moiety of NBSI intercalates into the binding pocket, stacking between the upper noncanonical base pair A15--G28 and the lower base triple U8--A29--C30 formed in the junction region J12, whereas the terminal (2-hydroxyethyl) (methyl)amino group of NBSI protrudes outwards from the binding pocket ( Figure 5E). C14 and G9 on the left side of the binding pocket further help anchor NBSI firmly in the binding pocket. Two hydrated magnesium ions (M1 and M2) participate in the tertiary folding of the NBSI-bound Clivia aptamer ( Figure 5E). M1 is located adjacent to the NBSI-binding pocket, where it coordinates hydrogen bonds with the 2′-OH of U8 and the O6 of G28 through hydrated water molecules ( Figure 5E). Although Clivia shows similar recognition to multiple NBSI-derived fluorophores compared with NBSI, molecular docking analysis of the binding pocket revealed that it employs a distinct fluorophore recognition pattern compared with other fluorescent RNA aptamers. *In vitro* fluorescence assays and live-cell imaging experiments in HEK293T cells further confirmed the orthogonal utilization of Clivia/NBSI and its derivatives in tracking diverse RNA molecules alongside other fluorescent RNA aptamer systems [91]. Moreover, the robust folding behavior of Clivia enables tailoring of the original Clivia motif to a minimal Clivia fluorogenic module, facilitating the design of multivalent Clivia fluorogenic aptamers containing tandem arrays of NBSI binding pockets. Compared with single Clivia fluorescent aptamers, these multivalent Clivia arrays display increased fluorescence in the presence of a controlled concentration of NBSI [91].

## Discussion

As mentioned above, many previously developed and structurally characterized fluorogenic RNA aptamers employ G-quadruplex tertiary folds, highlighting the importance of this structural motif for fluorescence activation. Each square planar platform of the G-quadruplex is formed by four noncanonical Hoogsteen hydrogen-bonding guanine bases. These structures provide highly stable, flat, hydrophobic platforms, which effectively restrain fluorophores into rigid planar or near-planar conformations. Moreover, these G-quadruplexes can accommodate other RNA functional groups, enabling additional hydrogen bonding interactions with the fluorophores to increase the affinity and selectivity of the fluorogenic aptamer. However, previous studies have shown that the formation of G-quadruplexes is specifically inhibited by helicases in bacteria, yeast, and mammalian cells [ 92‒ 95]. The misfolding tendency of G-quartet-containing aptamers has limited their broad application *in vivo* .

Notably, fluorescent RNA aptamers also employ base quadruples rather than G-quadruplexes, as exemplified by the MG aptamer and Pepper aptamer [ 56, 58]. In the recently reported fluorescent RNA aptamer Clivia, which adopts a non-G-quadruplex structural fold, three consecutive adenine-involved base triples are observed to stabilize the overall architecture and constitute the planar stacking platform for the ligand-binding pocket [57]. Similarly, A-minor base triplets have also been identified in the tertiary complex structure of the MG aptamer bound with TMR dye, suggesting that A-minor base triplets are likely to play an important role in the folding and stability of the RNA fluorogenic aptamer [ 58 , 59]. Investigations into the tertiary structure of these fluorogenic RNA aptamers highlight the inherent versatility of RNA molecules and demonstrate their ability to effectively recognize and bind to diverse fluorophore molecules.

The ideal characteristics of fluorescent RNA aptamers include but are not limited to, being monomeric, stable, bright and multicolored, which enables them to be powerful tools for visualizing RNA localization and transport in live cells. However, challenges such as weak cell brightness, poor photostability and low folding efficiency have hindered their widespread application [ 35, 36, 40, 66, 76]. The optimization and improvement of RNA aptamers have been achieved through structure-guided mutagenesis [ 66, 73], conventional SELEX screening combined with FACS or microfluidic-based technology [ 36, 44, 76, 96], and sequence rescreening from stable RNA scaffolds [40].

In addition, structural information on the ligand-binding pockets of fluorescent RNA aptamers also enables rational optimization or modifications of the cognate fluorophores, thereby expanding their spectral diversity and facilitating their application in RNA imaging [ 38, 39, 55, 57, 78]. For example, replacing the methyl substituent in DFHBI by a trifluoroethyl substituent resulted in DFHBI-1T, featuring redshifted excitation and emission spectra. Compared with DFHBI, DFHBI-1T results in lower background fluorescence and higher brightness when incubated with cells [78]. Similarly, HBI fluorophores have been modified to produce a series of derivatives with large Stokes shifts with yellow to red fluorescence emission, which were further reselected against the Chili aptamer [39]. The strategy yielded DMHBO ^+^, which binds to the Chili aptamer with low-nanomolar affinity. In addition, the substitution of the hydroxamic acid in DFHO with methyl acrylate in DFAME ^+^ results in redshifted emission, providing advantages for cellular imaging [38]. Additionally, a series of HBC analogues have been synthesized by tuning the aromatic π-structure or adjusting the electron donor and acceptor capabilities, expanding the spectral range of Pepper fluorescent RNA [55]. Similarly, NBSI, with a typical chromophore structure, has been further tuned by modifying the fluorophore structure to generate a series of derivatives with a broader range of fluorescence wavelengths, including a larger Stokes shift [57].

With advancements in diverse bright, photostable, and cell-permeable fluorophores and their corresponding fluorogenic RNA aptamers, as well as the accumulation of comprehensive structural information, the use of RNA molecules to track or image cellular RNA and drug RNA will be easily achieved. This progress has been significantly enhanced by the computer-aided rational design of fluorophores, which allows for precise tuning of their properties to meet specific experimental needs. The integration of advanced fluorophore technology with RNA research has greatly accelerated scientific discoveries and medical applications in RNA biology. In addition, these advancements also support the development of RNA-based therapeutics, offering new avenues for drug design and delivery.
